# Systematic evaluation of NIPT aneuploidy detection software tools with clinically validated NIPT samples

**DOI:** 10.1371/journal.pcbi.1009684

**Published:** 2021-12-20

**Authors:** Priit Paluoja, Hindrek Teder, Amin Ardeshirdavani, Baran Bayindir, Joris Vermeesch, Andres Salumets, Kaarel Krjutškov, Priit Palta

**Affiliations:** 1 Doctoral Programme in Population Health, Faculty of Medicine, University of Helsinki, Helsinki, Finland; 2 Institute of Clinical Medicine, University of Tartu, Tartu, Estonia; 3 Competence Centre for Health Technologies, Tartu, Estonia; 4 Institute of Biomedicine and Translational Medicine, Department of Biomedicine, University of Tartu, Tartu, Estonia; 5 Department of Human Genetics, KU Leuven, Leuven, Belgium; 6 Department of Obstetrics and Gynaecology, Institute of Clinical Medicine, University of Tartu, Tartu, Estonia; 7 Division of Obstetrics and Gynecology, Department of Clinical Science, Intervention and Technology (CLINTEC), Karolinska Institutet, Stockholm, Sweden; 8 Estonian Genome Centre, Institute of Genomics, University of Tartu, Tartu, Estonia; 9 Institute for Molecular Medicine Finland (FIMM), HiLIFE, University of Helsinki, Helsinki, Finland; University of Technology Sydney, AUSTRALIA

## Abstract

Non-invasive prenatal testing (NIPT) is a powerful screening method for fetal aneuploidy detection, relying on laboratory and computational analysis of cell-free DNA. Although several published computational NIPT analysis tools are available, no prior comprehensive, head-to-head accuracy comparison of the various tools has been published. Here, we compared the outcome accuracies obtained for clinically validated samples with five commonly used computational NIPT aneuploidy analysis tools (WisecondorX, NIPTeR, NIPTmer, RAPIDR, and GIPseq) across various sequencing depths (coverage) and fetal DNA fractions. The sample set included cases of fetal trisomy 21 (Down syndrome), trisomy 18 (Edwards syndrome), and trisomy 13 (Patau syndrome). We determined that all of the compared tools were considerably affected by lower sequencing depths, such that increasing proportions of undetected trisomy cases (false negatives) were observed as the sequencing depth decreased. We summarised our benchmarking results and highlighted the advantages and disadvantages of each computational NIPT software. To conclude, trisomy detection for lower coverage NIPT samples (e.g. 2.5M reads per sample) is technically possible but can, with some NIPT tools, produce troubling rates of inaccurate trisomy detection, especially in low-FF samples.

## Author summary

Non-invasive prenatal testing analysis relies on computational algorithms that are used for inferring chromosomal aneuploidies, such as chromosome 21 triploidy in the case of Down syndrome. However, the performance of these algorithms has not been compared on the same clinically validated data. Here we conducted a head-to-head comparison of WGS-based NIPT aneuploidy detection tools. Our findings indicate that at and below 2.5M reads per sample, the least accurate algorithm would miss detection of almost a third of trisomy cases. Furthermore, we describe and quantify a previously undocumented aneuploidy risk uncertainty that is mainly relevant in cases of very low sequencing coverage (at and below 1.25M reads per sample) and could, in the worst-case scenario, lead to a false negative rate of 245 undetected trisomies per 1,000 trisomy cases. Our findings underscore the importance of the informed selection of NIPT software tools in combination with sequencing coverage, which directly impacts NIPT sequencing cost and accuracy.

This is a *PLOS Computational Biology* Benchmarking paper.

## Introduction

Non-invasive prenatal testing (NIPT) is widely used and enable highly accurate fetal chromosomal aneuploidy screening [1]. As NIPT relies on whole-genome sequencing (WGS) or targeted sequencing of cell-free DNA (cfDNA) extracted from the peripheral blood samples from the pregnant woman, it reduces the number of invasive fetal testing procedures [2,3]. Computational analysis of the resultant sequencing data is used to detect excess or deficient sequencing reads within specific chromosomes and thereby provides clinical information regarding possible aneuploidy of the fetus [3,4].

To date, several computational NIPT analysis tools for WGS-based NIPT have been examined in the literature. These include GIPseq [3], NIPTmer [5], NIPTeR [6], RAPIDR [7], DASAF R [8], Wisecondor [9], and WisecondorX [9,10]. However, while these computational tools are commonly used, no head-to-head evaluation studies of these NIPT tools on the same clinically validated samples is available.

Computational NIPT studies have indicated that the most critical reliability factor of NIPT analysis is sequencing depth, also known as read coverage [10,11]. A higher sequencing depth represents a greater likelihood of comprehensive genome-wide interrogation and more evidence for the detection of consecutive chromosome-spanning gains or losses, thus improving diagnostic sensitivity to aneuploidies [11]. Currently, a sequencing depth of 10M reads per sample (RPS) is considered to be sufficiently reliable for clinical screening for risk of Down syndrome, Edwards syndrome, and Patau syndrome [11].

A second relevant aspect of the computational NIPT aneuploidy detection is the analytical interpretation of the computational tool output. Commonly used NIPT tools output a per chromosome metric describing the difference (or similarity) of the sample of interest compared to reference group samples, representing the NIPT data of known/validated euploid samples [3,5,6,9,10]. While some tools do provide explicit guidelines for interpreting the output [6], in general, the NIPT software output and analytical interpretation are not well standardised and tend to be highly dependent on the software, laboratory protocols, sample pre-processing and also reference group utilised in the process. A universally usable framework for the interpretation of these metrics is required.

Finally, it is relevant to consider that in the NIPT data analysis, sequencing reads originate both from the studied fetus/placenta and the mother [12]. The maternal chromosomal status can be considered as a baseline and the fetal chromosomal status as the signal of interest [13]. The proportion of fetal DNA fraction (FF), generating the signal of interest, is a critical sample-level quality control determinant, allowing to detect samples with too low FF, for which neither the computational aneuploidy calling nor euploidy confirmation could be performed with confidence. Low-coverage WGS-based NIPT assays and subsequent computational analyses do not distinguish whether sequencing reads are fetal or maternal origin, thus a sufficient proportion of FF is crucial to obtain a true result [3,5,6,9,10,13].

In this study, we have assessed these critical aspects of NIPT and their effect on the accuracy of five commonly used NIPT software tools. We systematically evaluate the published computational NIPT tools’ performance and accuracy on a set of clinically validated samples, considering various sequencing depths and the proportion of cell-free fetal DNA (FF). We define and validate a straightforward and universal Z-score quantile cut-off based framework that can be unambiguously used to describe and compare aneuploidy calling software tools.

## Results

We compared the results of five NIPT computational software tools on the same set of clinically validated NIPT samples. By systematically subsampling sequencing reads of studied samples (to artificially lower their sequencing coverage) and by analysing the chromosomal Z-scores obtained with different NIPT software tools, we determined their ability to detect known trisomies and confirm euploid samples. We determined the lower sequencing depth threshold for each software, while also considering FF in analysed samples.

### Sequencing depth effect on aneuploidy detection

**Fig 1:**
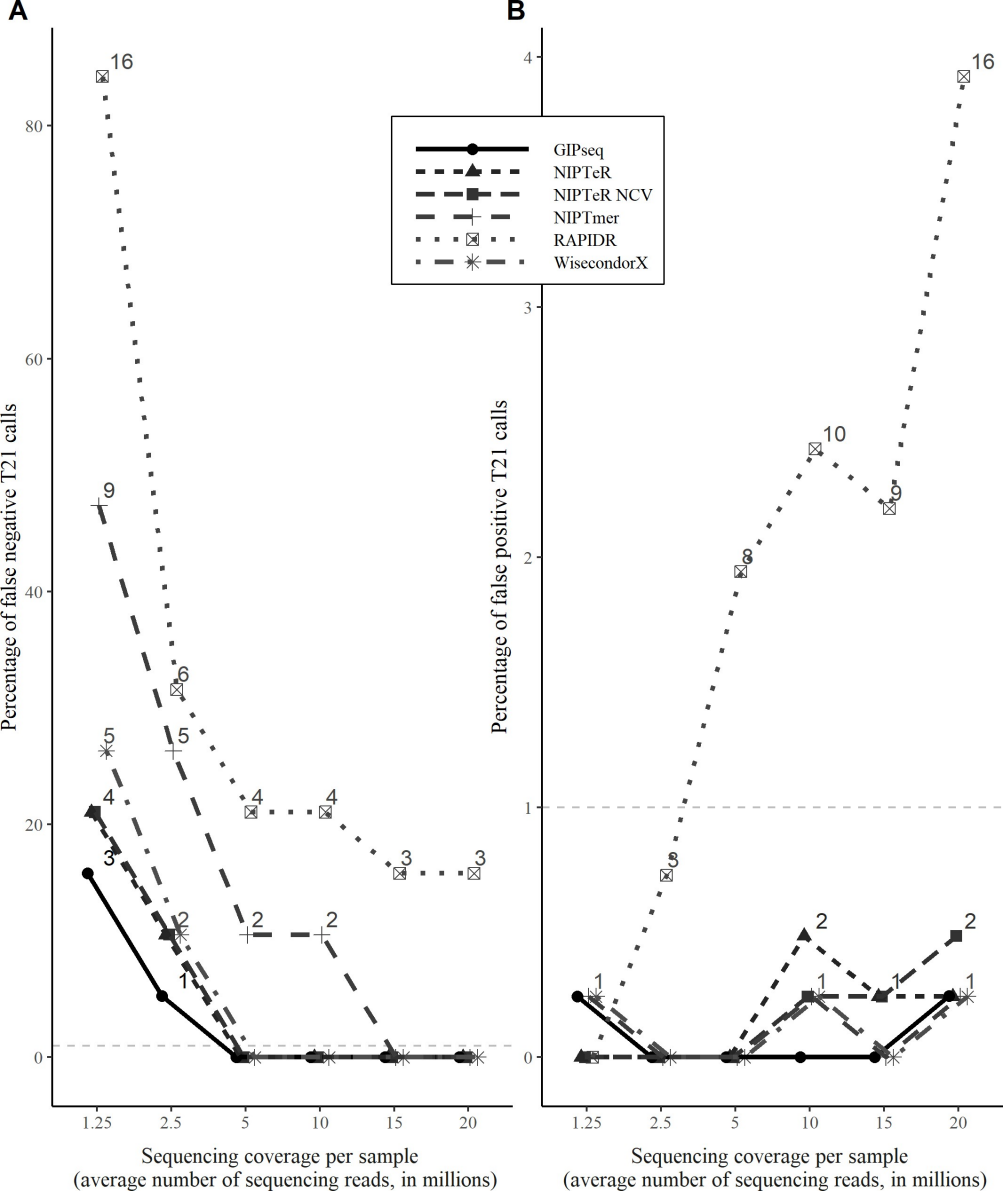
Trisomy detection accuracy of tested NIPT software tools across different sequencing depths. (A) Percentages and absolute numbers of undetected trisomy cases among known trisomies. (B) False-positive T21 results among known euploid samples. The horizontal dashed line in each graph marks the 1% cut-off level often used in clinical screening.

The numbers of true and false trisomy findings obtained with each software across a range of sequencing depths were collated with a uniform empirically defined Z-score Z_e_ threshold for each NIPT software (see Materials and Methods). The trisomy 21 (T21), trisomy 13 (T13), and trisomy 18 (T18) detection accuracy rates obtained for each of compared tools (GIPseq, NIPTeR, NIPTer NCV, NIPTmer, RAPIDR, WisecondorX) are shown in **Figs 1** and **S1** and **S2**, respectively.

**Fig 2:**
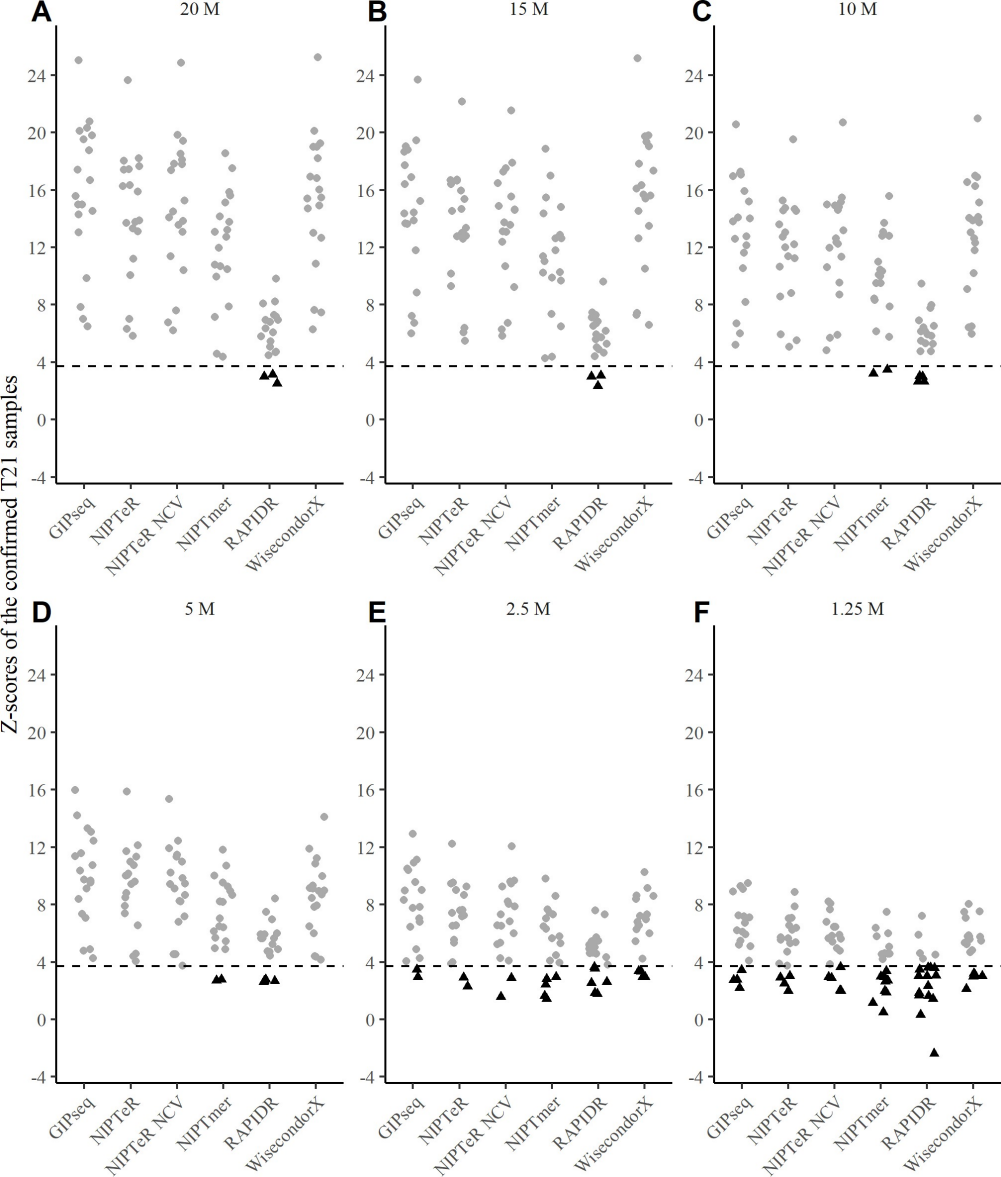
Z-scores of clinically validated T21 samples across a range of sequencing depths. Z-scores of known trisomy samples at sequencing depths of 20M RPS (A), 15M RPS (B), 10M RPS (C), 5M RPS (D), 2.5M RPS (E), and 1.25M RPS (F) are shown. Undetected (false negative) trisomies falling below the *Z*_*t*_ cut-off thresholds (black dashed line in each graph) are represented as black triangles.

We observed mostly accurate outputs with sequencing coverages above 5M RPS (i.e., read per sample), whereas differentiation trends among compared algorithms became apparent at lower sequencing depths, particularly with respect to false-positive and false-negative trisomy calls. Furthermore, all algorithms demonstrated a considerable increase in the number of false-negative trisomy findings below 5M RPS (**Fig 1** and **S1 Table**). These changes in trisomy detection accuracy were driven by more conservatively estimated Z-scores, which decrease systematically at lower sequencing depths, as shown for T21 in **Fig 2**.

Interestingly, chromosome-specific differences in accuracy were observed between the NIPTeR and NIPTeR-NCV. Although both detected T21 cases equally accurately, the latter showed better T18 detection accuracy (**S2 Fig**), especially in lower sequencing coverage conditions. GIPseq, NIPTeR, and WisecondorX performed similarly well at sequencing depths of 10M RPS and higher, followed by NIPTmer and RAPIDR. The GIPseq, WisecondorX, and NIPTeR algorithms also yielded similar accuracies at sequencing coverages lower than 10M RPS and produced very similar results for T21 and T18 detection. Furthermore, our results demonstrate that the GIPseq, WisecondorX, and NIPTeR tools provide reasonably good accuracy for trisomy detection, even at very low sequencing depths (2.5M and 1.25M RPS). The above results demonstrate that sufficient read coverage is required for accurate NIPT aneuploidy inference.

In addition to considering how sequencing depth alters the reference panel driven changes in Z-score variability and increased uncertainty (especially at lower coverages), it is also relevant to consider how lower sequencing depth affects naturally occurring arbitrary sequencing read placement and, consequently, uncertainty in the studied sample’s Z-score estimation. In corresponding analyses (done with NIPTeR, see Materials and Methods), we observed that having a sequencing coverage of 7M RPS or less can affect arbitrary sequencing read placement considerably and thus impair trisomy detection accuracy. Corresponding simulations with clinically confirmed trisomy cases (**S3 Fig**) and euploid samples (**S4 Fig**) demonstrated that a proportion of trisomy cases are likely to become undetectable if an aneuploidy calling algorithm does not consider this additional source of uncertainty, especially at sequencing depths below 7M RPS. Therefore, appropriate correction methods are required for confident use of computational NIPT analytical tools under conditions of very low sequencing coverage.

While working with the Z-scores for the euploid reference group that were used to determine the empirical trisomy calling cut-off of *Z*_*e*_, we observed empirical *Z*_*e*_ scores changes with some tools and coverages. Importantly, these cut-offs have a direct effect on the number of false-negative trisomy cases. For example, the WisecondorX *Z*_*e*_ threshold for T21 varied from 3.14 to 4.16, depending on sequencing depth (**S5 Fig**). With the theoretical cut-off *Z*_*t*_, the WisecondorX introduces two additional (total of four) undetected trisomies at a 2.5M RPS sequencing depth (**Figs 1** and **2E**), as compared to the hereby suggested usage of empirically derived (reference data variance aware) threshold of *Z*_*e*_. This observation suggests that NIPT software tools can benefit from *Z*_*e*_ cut-off calibration as a universal uniform cut-off *Z*_*t*_ does not consider differences between various NIPT tools and analysed data (which can also harbour laboratory-specific effects), leading to false-negative and false-positive trisomy predictions.

### Accuracy of fetal DNA fraction estimation at low sequencing depths

**Fig 3:**
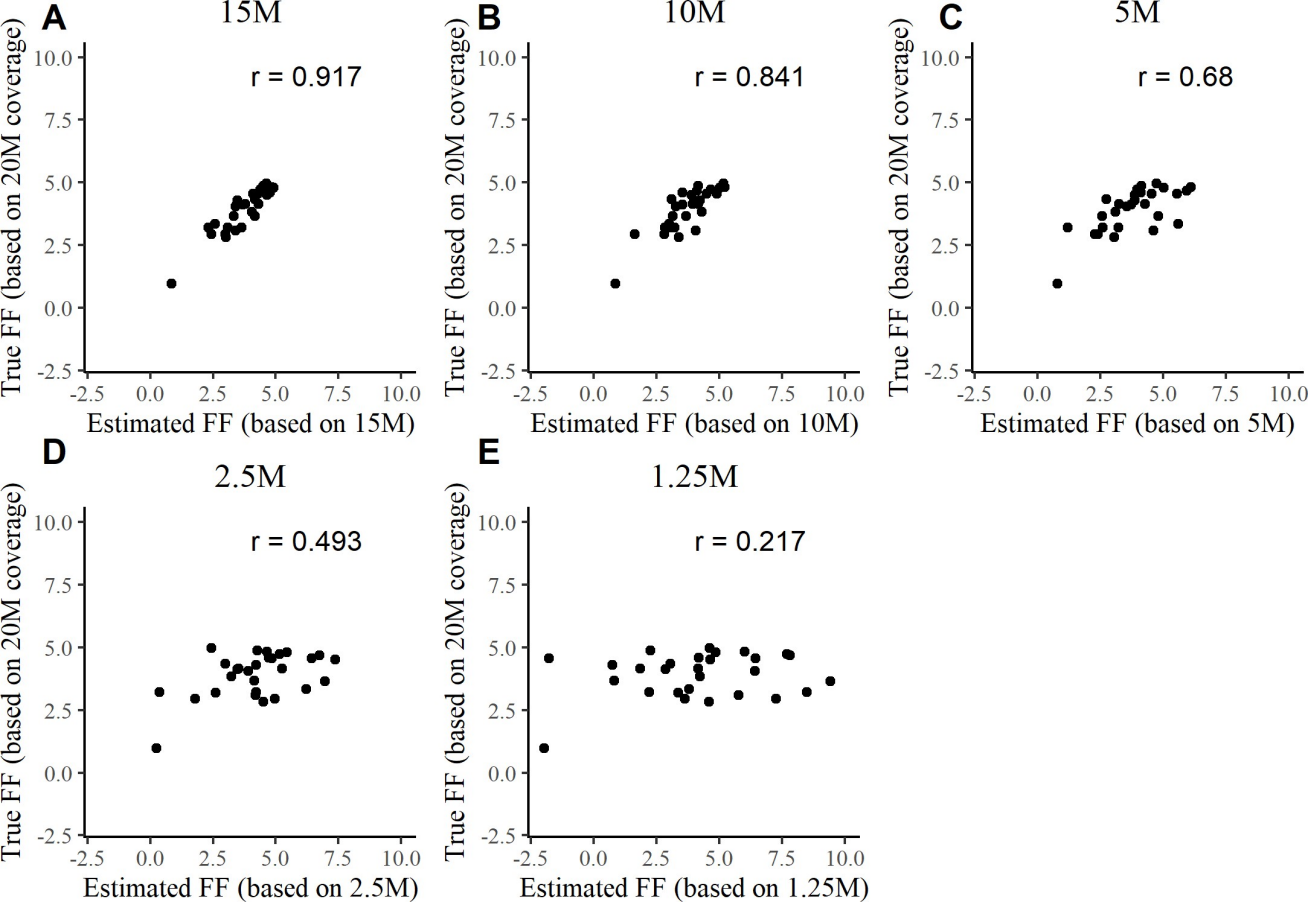
Correlation between the ‘true’ (based on 20M RPS data) and low sequencing depth based FF estimates. Pearson correlation data are shown for 20M RPS FF 0–5% estimates and estimates obtained at sequencing depths of 15M RPS (A), 10M RPS (B), 5M RPS (C), 2.5M RPS (D), and 1.25M RPS (E).

Ideally, FF estimates would be consistent for a sample regardless of sequencing depth. However, we observed that FF estimates at lower sequencing depths were inconsistent with FF estimates obtained in the reference coverage condition (~20M RPS) (**Figs 3** and **S6**). For FFs in the range of 0–5%, the Pearson correlation value obtained between 1.25M RPS FF estimates and corresponding 20M RPS FF estimates was only 0.217 (**Fig 3E**). For the same samples, the FF correlations were relatively consistent at higher sequencing depths. For example, the correlation value obtained between 10M RPS FF and 20M RPS FF estimates was 0.841 (**Fig 3B**). At higher FF values (true FF, 5–15%), sequencing depth had a more subtle influence. For example, we obtained a robust Pearson correlation value of 0.959 between 10M RPS FF and 20M RPS FF estimates, but a Pearson correlation value of only 0.636 between 1.25M RPS FF and 20M RPS FF estimates (**S6 Fig**).

**Fig 4:**
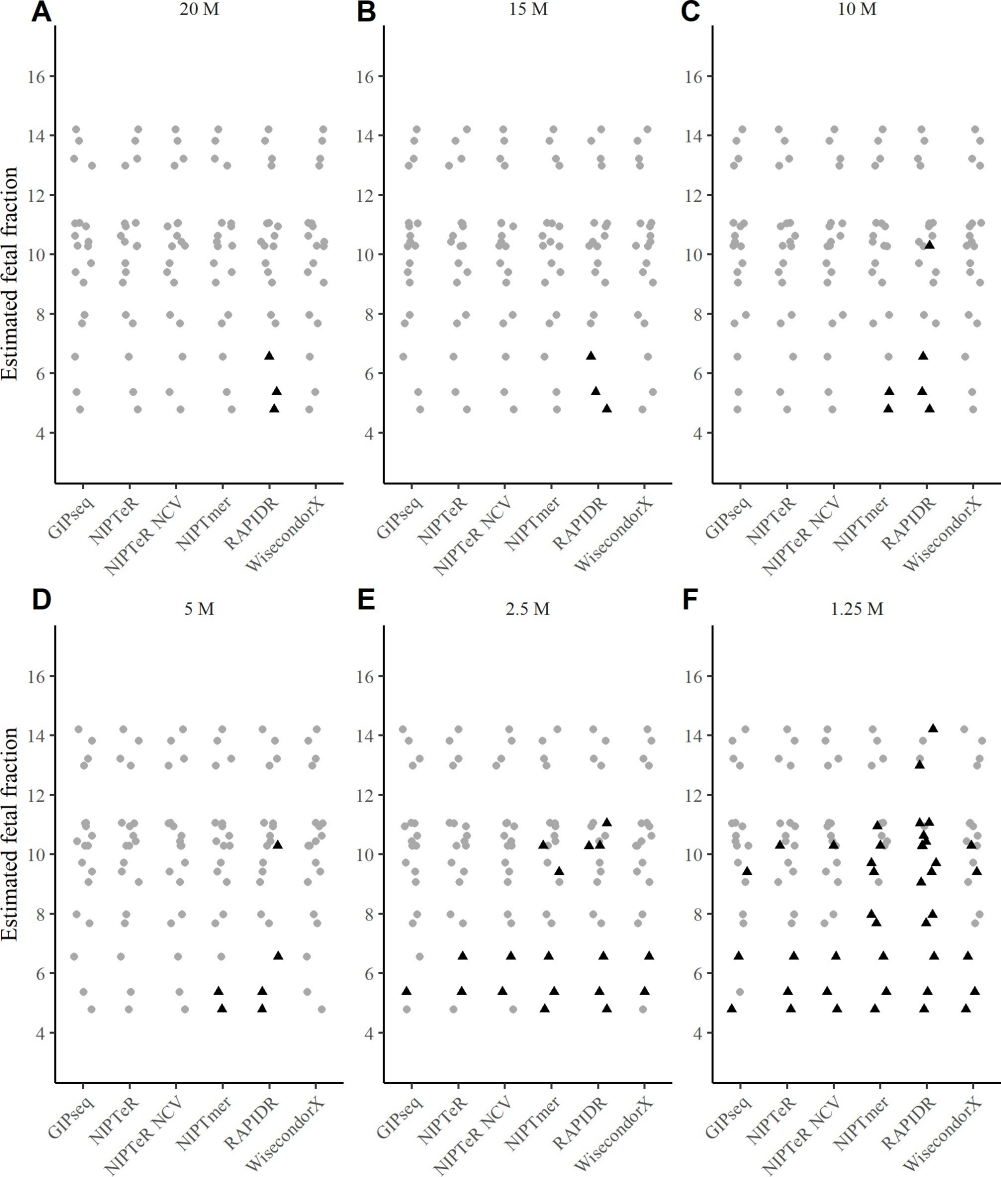
The effect of FF on detection of T21 across different sequencing coverages. *Z*_*e*_ cut-off was used for identifying the presence of the trisomy (internal classification in the case of GIPseq). Black triangles represent undetected trisomy cases. The 20M RPS group served as the standard for FF calculations. Data obtained with sequencing depths of 20M RPS (A), 15M RPS (B), 10M RPS (C), 5M RPS (D), 2.5M RPS (E), and 1.25M RPS (F) are shown.

The presently observed patterns of correlations between FF estimates at low sequencing depths and FF estimates at higher sequencing depths suggest that FF may be systematically over-estimated under low-sequencing-depth conditions. This risk may beget faulty overconfidence when determining aneuploidy/euploidy status at low sequencing depths. For example, in a scenario where a sample with a very low FF (which should, in clinical applications, fail FF-based quality control) is assayed at a low (e.g., ~5M RPS) sequencing coverage, false confidence may be given to the result regarding chromosomal euploidy or trisomy classification. Our further analysis confirmed this possibility in assays with low sequencing depths (**Figs 4 and S7 and S8**). Notably, in the case of the lowest sequencing depth analysed (1.25M RPS), all T21 samples with a FF below 7% (except one analysed by GIPseq) returned an incorrect normal euploid result (**Fig 4F**). At a sequencing depth of 2.5M RPS, all tested software tools failed to detect at least one (out of 19) case of T21 (**Fig 4E**). At sequencing depths of 5M RPS and higher, majority of the software tools detected most T21 cases correctly, with the only exceptions being NIPTmer and RAPIDR, which failed to identify two and four (out of 19) trisomies, respectively.

Interestingly, GIPseq resulted in one undetected T21 case at 2.5M RPS, yet it was detected at 1.25M RPS (**Fig 4E** and **4F**). At the same time, there was another sample at 1.25M RPS detected by GIPseq but not with any other software (**Fig 4F**).

The present results demonstrate that accurate trisomy detection at low sequencing depths is affected substantially by the computational tool selected as well as by the accuracy of estimated FFs. NIPTeR NCV (see NIPTeR description in Materials and Methods) and WisecondorX were the only computational tools tested that had no false negative trisomy results with an extremely low FF (3.65%) at a 5M RPS sequencing depth (**Figs 1, 4** and **S1, S2, S7** and **S8**). With a 3.65% FF, GIPseq and NIPTeR had no false negative trisomy calls at 10M RPS, whereas NIPTmer only achieved a no-false-negatives outcome with a 3.65% FF when a sequencing depth of 20M RPS was used. We found that RAPIDR analyses missed some trisomy cases at all tested sequencing coverages (**Table 1**).

**Table 1:** The key considerations for each of the compared algorithms. Table covers minimal coverage where the computational tool could simultaneously detect trisomy cases in chromosomes 13, 18 and 21 with less than 1% undetected and false-positive trisomy calls, minimal fetal fraction from which upwards there are no undetected T13, T18 and T21 calls, and software tool usability.

Software	Clinically usable coverage with less than 1% of false negative trisomy calls (M RPS) *	Clinically usable coverage with less than 1% of false positive trisomy calls (M RPS) **	Lower fetal fraction threshold at 5 M RPS, no false negatives	Lower fetal fraction threshold at 10 M RPS, no false negatives	Usability
GIPseq	10	1.25	4.8%	3.7%	Input: FASTQ Available: not publicly available
NIPTeR (Z-score)	10	1.25	4.8%	3.7%	Input: BAM Reference genome: GRCh 37, 38 Available: CRAN, GitHub Usability: completely R-based package, requires scripting in R Used user-manual: vignette-based (includes examples) Reference creation, usage: straightforward
NIPTeR (NCV score)	5	1.25	3.7%	3.7%	See NIPTeR (Z-score)
NIPTmer	20	1.25	6.6%	6.6%	Input: FASTQ Reference genome: GRCh 37, 38 Available: URL Usability: requires scripting in bash to set up, no examples, uses different programming languages, tools and scripts. Used user-manual: readme Reference creation, usage: moderate due to no examples
RAPIDR	T21: 15.8% FN calls on ≥ 15M RPS T18: all trisomies missed on 1.25–20 M RPS T13: 33.3% FN calls on ≥ 10M RPS	2.5	T21: 10.4% T18: all missed T13: 8.5%	T21: 10.4% T18: all missed T13: 5.9%	Input: BAM Reference genome: GRCh 37 Available: Anaconda, CRAN, GitHub Usability: completely R-based package, requires scripting in R Used user-manual: vignette-based (includes examples) Reference creation, usage: straightforward
WisecondorX	5	1.25	3.7%	3.7%	Input: NPZ from BAM Reference genome: GRCh 37, 38 Available: Anaconda, GitHub, PyPI Usability: Python-based command-line tool, requires a little knowledge of bash Used user-manual: GitHub readme with examples Reference creation, usage: straightforward

* In the tested sequencing depth range, RAPIDR software always produced more false negatives than 1%.

** Trisomy calling becomes more conservative when sequencing coverage decreases (**Fig 2**), resulting in fewer false-positive trisomy calls.

## Discussion

Non-invasive prenatal testing (NIPT) is an effective screening method for fetal aneuploidy testing, which is based on laboratory and computational analysis of cell-free DNA derived from the peripheral blood of pregnant women. Hence, a correctly set up NIPT assay allows reducing invasive procedures while still enabling the detection of fetal aneuploidies at high confidence. Although there are several computational NIPT tools for WGS-based NIPT, there are no published comparisons of these tools, which would allow NIPT laboratories to select an optimal analytical software matching their NIPT sequencing assays.

As expected, we found that the currently publicly available computational tools can accurately detect chromosome 13 trisomy (causing Patau syndrome), trisomy 18 (Edwards) and trisomy 21 (Down) accurately at 5M RPS and higher sequencing depths (**Table 1**). Furthermore, our data demonstrated that a well-chosen computational NIPT software employed in combination with appropriate Z-score thresholds can be used at lower sequencing coverages, even at sequencing depths below 5M RPS. This is in line with the current knowledge, as NIPT usage potential at lower coverages (such 2.2M RPS) has been demonstrated previously [14]. However, at lower sequencing depths (below 5M RPS), the compared computational tools yielded trisomy risks with differing accuracies, reflected by mostly with a systematically increasing numbers of false-positive and false-negative trisomy cases (**Figs 1** and **S1** and **S2**). Detection capability differences among the algorithms were most evident with very low read coverages, such as 1.25M RPS, at which one algorithm detected 13 more cases of trisomy than another (**Fig 1**). Furthermore, at very low sequencing depths, it is particularly important to consider how uncertainty derived from naturally occurring arbitrary sequencing read placement affects sample Z-score inferences, and thus the risk of undetected trisomies (**S3 Fig**). Notably, at a sequencing depth of 2.5M RPS, our results suggest that use of the least accurate algorithm would result in missed detection of about a third of trisomies (~316/1,000 trisomic pregnancies tested) as well as some (~7/1,000) false positive trisomy results for euploid fetuses. Additionally, if not corrected for, the uncertainty in Z-score inference associated with 2.5M RPS coverage would introduce a near 1-in-7 false negative rate (139 undetected trisomies per 1,000 trisomy cases).

The compared algorithms were found to have differing sensitivities to a very low FF (**Figs 4** and **S7** and **S8**). In general, low sequencing depths coupled with low FFs further decreased trisomy detection accuracy (**Figs 4** and **S8**). That said, there are NIPT algorithms that work accurately with a FF lower than 4% [15]. Koc *et al*. also observed that NIPT test failures (i.e., no result) are often related to a low FF with a low sequencing coverage [14]. Moreover, depending on the software used for FF calculation, FF estimator accuracy can fall at low sequencing coverages in cases of truly low FFs (**Figs 3** and **S6**). Similar results were obtained by Miceikaitė *et al*., who demonstrated that FF estimator accuracy tends to decrease at low sequencing depths [16]. Severe FF estimation inaccuracies can lead to the inappropriate analysis of a very low FF sample, which should fail an FF-based quality control. In such cases, the aneuploidy/euploidy outcome would be associated with false confidence. Although not tested and analysed comprehensively, our results suggest that for samples with a FF ≥ 7%, the use of several computational NIPT tools as an ensemble (to supplement sensitivity across conditions) may provide perfectly accurate aneuploidy detection at 5M RPS (**Fig 4**) as well as higher accuracy at 2.5M RPS than the strategy of using a single ‘best’ NIPT software tool.

The most relevant difference among the tested algorithms was the trisomy detection accuracy observed at 5–15M RPS. Regarding criteria such as accuracy with low coverage (5M RPS), availability, and licencing, NIPTeR and WisecondorX are similar, with differences only in licencing and output format (**Table 1**). WisecondorX also generates output files and figures for detected putative copy number variants. Conversely, the NIPTeR licence permits commercial uses that are restricted by the WisecondorX licence. It should further be considered that WisecondorX benefits from the use of *Z*_*e*_ thresholds (**S5 Fig**), and NIPTeR NCV performs T18 detection more accurately than NIPTeR (**S2 Fig**). Our results affirm that both NIPTeR and WisecondorX are great default choices for computational NIPT analysis.

Regarding limitations, it should be noted that while our study had a sufficient quantity of NIPT samples to compose a representative NIPT reference panel, we had a relatively limited number of clinically validated trisomy samples. Although there is no reason to expect that adding trisomy samples would alter the accuracy patterns of the compared tools, future research could benefit from similar comparisons with increased numbers of clinically validated trisomy samples, ideally samples that were sequenced at lower coverages. Secondly, although we tried to eliminate all possible sources of technical bias, the artificial downsampling of samples (to lower RPS) might not provide a perfect reflection of the natural read placement variability in samples natively sequenced at lower coverages, the outcomes of which could vary slightly from our results. That said, a similar downsampling approach was implemented successfully by Miceikaitė *et al*. in their investigation of FF estimation accuracy at low sequencing depths [16]. Our read placement variability effect analysis on Z-score inference was carried out only with the NIPTeR software, one of the most accurate tools in our comparison. In research and clinical practices utilising WGS-based NIPT with low sequencing coverage, similar analyses should be considered with any algorithm used for trisomy detection. Similar actual (non-downsampled) sequencing data based analyses or simulations can be carried out with any aneuploidy detection algorithm to assess the exact magnitude of this phenomenon.

A well-designed NIPT assay protocol helps to minimise invasive procedures while enabling confident detection of fetal aneuploidies. In the present work, we have provided the first direct comparison, to our knowledge, of multiple computational NIPT tools for WGS-based NIPT, the findings of which are useful for enabling NIPT laboratories to select an optimal analytical software for their NIPT sequencing assays. The present work provides insights into WGS-based NIPT accuracy in relation to computational tool choice. Our results underscore the substantial influence that the computational tool selected for NIPT analysis and sequencing coverage have on the accuracy of trisomy detection, with divergence among NIPT tools becoming particularly pronounced at very low sequencing depths.

## Materials and methods

### Ethics statement

This study was performed with the written informed consent from the participants and with approval of the Research Ethics Committee of the University of Tartu (#315/T-13).

### Studied samples

Two sets of samples were used. **For the reference sample set**, a total of 669 known euploid samples were used. Of those, 326 of these samples were of female fetus pregnancies and 343 of male fetus pregnancies. All 669 samples had been reported previously to be euploid by the NIPTIFY screening test and postnatal evaluation. **The validation sample set** was based on a previously published validation study by Žilina *et al*. [17], consisting of 423 samples, of which 258 were high-risk pregnancies that had undergone diagnostic invasive prenatal analysis [17]. These included 19 samples with confirmed fetal chromosome 21 (T21), eight chromosome 18 (T18) and three chromosome 13 (T13) trisomy cases.

All samples were sequenced with Illumina NextSeq 500 platform, producing 85 bp single-end reads with an average per-sample coverage of 0.32× at the University of Tartu, Institute of Genomics Core Facility, according to the manufacturer’s standard protocols, as described previously [17].

### Sample pre-processing

For the alignment-based computational NIPT methods, each sequenced sample was aligned against human reference assembly GRCh37 (RAPIDR) or GRCh38 (NIPTeR, WisecondorX) depending on the software prerequisite. Next, the aligned sample was sorted, and the reads originating from a single fragment of DNA were marked as duplicates. For the k-mer based computational workflow NIPTmer, no special pre-processing was applied.

Both the validation set and the reference set were emulated to lower sequencing coverage. For this, each sample was subsampled into six different groups of subsamples. The average million reads per sample targets were: 20M, 15M, 10M, 5M, 2.5M, and 1.25M. For 5-20M, the lower sequencing coverage was emulated by leaving the appropriate number of NextSeq 500 output lanes out. For lower and equal to 2.5M, one lane (5M) was taken and subsampled with samtools view and then converted to FASTQ with samtools bam2fq [18], and the exact resulting coverage was then calculated for each sample. Clinically validated T21, T18, and T13 sample group information with the reference population and low-risk validation sample group are presented in **S2 Table**.

### Study approach

Computational NIPT methods applied in this study use a euploid reference set. For each analysed sequencing coverage, a corresponding reference set was created. For example, for the 5M RPS coverage, both the validation and reference samples were subsampled to 5M RPS analysed as a 5M RPS group. For each sample, coverage, fetal DNA fraction and Z-score estimates for chromosomes 13, 18, and 21 were calculated.

### Sequencing depth effect of aneuploidy detection

To analyse the effect of sequencing depth (read coverage) on aneuploidy detection, we systematically subsampled raw sequencing read data from the average of 20M reads to 1.25M reads per sample. Next, we applied a computational NIPT tool (with the reference corresponding to the RPS) to infer sample Z-scores. Then, we calculated the accuracy corresponding to different sequencing coverage for each software by counting the number of correctly or incorrectly detected known trisomies and euploid samples by comparing the sample Z-score with the empirically calculated cut-off *Z*_*e*_ (except for GIPseq, which was evaluated by the interpretations received from the GIPseq authors). We also found that NIPTeR, NIPTeR NCV, and RAPIDR do not provide results if the sequencing coverage is lower than 1.25 M reads per sample.

### Low sequencing coverage driven uncertainty in Z-score inference for trisomic and euploid samples

We used ten low-risk samples to determine how low sequencing depth and consequent arbitrary sequencing read distribution and binning affect the uncertainty in Z-score estimation. These samples were selected to have a high fetal fraction (FF estimates between 10.23–18.57%) and high sequencing read counts (read count between 22M–30M) and with chromosome 21 Z-scores close to zero (original read coverage NIPTeR Z-scores between -0.0996 and 0.0942). The BAM files of those samples were concatenated and sorted, leading to a single pooled low-risk sample with 247M reads. The concatenated sample was then randomly subsampled 2,000 times to groups of 2.5, 5, 10 and 20M RPS, followed by the NIPTeR Z-score calculations. The expected Z-score of each generated sample is approximately zero. Next, the deviations were found by subtracting the average group Z-score from the calculated Z-scores. These normally occurring deviations from the expected Z-score of 0 were added to the original validation sample Z-scores, leading to 2,358,000 simulated low-risk Z-scores and corresponding counts of false positive (FP) and true negative (TN) trisomy cases (based on *Z*_*t*_). Similarly to low-risk samples, the same methodology was also applied to T21 samples. The concatenation was done with eight T21 samples (read count 20M–33M, FF 10.43%–14.21%, chromosome 21 NIPTeR Z-score 13.77–23.64) that led to 203M RPS. For this simulation, 6, 7, 8 and 9M RPS reference group was created and similarly to 1.25 and 2.5M RPS, 20M RPS samples were downsampled.

### Sequencing depth effect on fetal DNA fraction estimation

To analyse the effect of sequencing depth on FF estimation, we first estimated FF for the 20M RPS group. Due to a limited number of samples with low FF estimations (12 samples with FF of 0–4%), we divided samples into FF groups of 0–5% (n = 28) and 5–15% (n = 302) for more accurate correlation estimates on samples with low FF. Next, we systematically subsampled raw sequencing read data from the average of 20M reads to 1.25M reads per sample. Finally, we compared the FF estimates of different sequencing coverages (also considering the FF group) with 20M by calculating Pearson correlation.

### Aneuploidy detection with empirically defined Z-score thresholds

All evaluated computational NIPT tools provided Z-score output for assessing the risk for aneuploidy. Each of the algorithms also has specific differences. For example, RAPIDR outputs trisomy calls [7], WisecondorX creates log_2_ ratio chromosome figures between the ratio of the observed number and expected number of reads [10], NIPTmer publication defines cut-off at Z-score of 3.5 [5], GIPseq provides algorithmic decision tree-like interpretation of the results [3], and NIPTeR avoids defining clear trisomy call cut-off [6]. However, to compare different algorithms’ performance on similar grounds, we defined a generally usable framework for Z-score calculations and comparisons, relying on a straightforward percent point (quantile) function. Specific quantile point allows specifying the cut-off value of the computational NIPT Z-score such that the probability of the euploid sample Z-score being less than or equal to the cut-off equals the chosen probability. This calculation can be done with the presumption that the mean and SD of the euploid sample group Z-score distribution is 0 and 1 (standard normal distribution) or with empirically observed mean and SD of the euploid sample reference group Z-score distribution. The first threshold is referenced as a (universal) theoretical cut-off *Z*_*t*_ in the calculations and the latter as the empirical cut-off *Z*_*e*_. A probability of 0.9999 was used for all the performed analyses: P_99.99_ (P[Z_sample_ ≤ z_cut off threshold_]) = 0.9999. Theoretically, given an informative NIPT assay data with sufficient coverage and a fetal fraction, it is expected to get one false-positive trisomy call per every 10,000 analysed normal euploid samples.

### Compared software tools

For a comparison between different tools, the Z-score was used for scoring as all the computational NIPT tools supported it in the analysis. Instead of the older Wisecondor software, we decided to use the newer WisecondorX. Also, DASAF R [8] is made available by the authors, but the corresponding web links in the publication to the software are inoperable, and the software was not included. Execution and the analysis of the output of the tools were done by scripting in WDL, Python, R, and Bash in the computer cluster of the High Performance Computing Center of the University of Tartu [19]. If the tool failed to operate on low coverage, then analysis of that coverage for the failed tool was left out. The most relevant data-analysis procedures, parameters, and analysis aspects for each software are shortly described and discussed in the paragraphs below.

### NIPTeR

For NIPTeR, v1.0.2 was used. In the reference group’s creation, each sample was binned with *bin_bam_sample* (parameter *separate_strands* set to *TRUE*), then GC corrected (method *gc_correct*, parameters *method* set to *‘bin’*, *ref_genome* set to *‘hg38’*, and *include_XY* set to *FALSE*). After that, all the GC-corrected samples were marked as the control group. No control group matching was done as all the tested tools had to have the same control group. For each validation sample, the sample was binned and GC corrected. For scoring, each sample was chi corrected (*chi_correct(sample*, *control_group)*), and after that, the Z-score was calculated with the function *calculate_z_score*. Additionally, as NIPTeR supports normalized chromosome value (NIPTeR NCV), which minimises variation between sequencing runs, NCV results were also analysed (calculated with *prepare_ncv(max_elements = 9)* and *calculate_ncv_score*) and compared [6,20].

### WisecondorX

Wisecondor (Paco_0.1) was tested on a 5M RPS group using the quick start guide published on their official GitHub page. Due to the Wisecondor output not containing a single Z-score for the chromosome but multiple Z-scores for the chromosome regions, Wisecondor was left out from the further analysis due to the incomparability with the other tested software metrics and the existence of WisecondorX, which provides a Z-score for the entire chromosome. For WisecondorX, v1.1.5 was used. Pre-processed samples were converted to .npz format with *WisecondorX convert* command. Next, the reference was created with *WisecondorX newref—nipt—refsize 669—binsize 100000* directive. For Z-scores, the *chr_statistics* file from the output was used after applying the *predict* command.

### NIPTmer

NIPTmer binaries were obtained from the University of Tartu Department of Bioinformatics webpage. Pre-built lists, which were packaged with the software, were used. Although the cleaned lists were made with the GRCh37 reference genome, since it is not an alignment-based method and GRCh37 lists are known to work [17], the GRCh37 version was kept. Binaries were updated to the latest version due to software issues with the packaged binary.

### GIPseq

The GIPseq [3] was run at the KU Leuven. The samples were uploaded to the KU Leuven Google Cloud bucket and analysed by GIPseq. The raw output of the analysis was shared with the authors. The GIPseq NIPT pipeline provides a sophisticated output, including quality scores and analysability for each sample. They also define specific decision rules for calling each sample state and have more states than euploid or aneuploid, including monosomy, segmental or undetermined. Since most of the other computational NIPT tools used in the analysis (I) provide only Z-score output, (II) do not have a pre-defined cut-off threshold, (III) do not provide more scores than Z-scores, the GIPseq pipeline is not directly comparable with other computational NIPT tools. For assessing the number of false-negative and false-positive trisomy cases and the effect of fetal fraction, GIPseq authors interpretations (euploid or trisomy) for each sample were used. For assessing the effect of the average sample read count for Z-scores, the theoretical Z-score cut-off was used for comparability between different computational tools.

### RAPIDR

For RAPIDR, v0.1.1 was used. Since RAPIDR requires, according to the manual GenomicRanges of version 1.14.4, which is challenging to acquire due to its old release date and RAPIDR will not work with later versions, a workaround was found, allowing to use RAPIDR 0.1.1 with most recent R packages. For this, R package GenomicAlignments (bioconductor-genomicalignments 1.22.0) was installed and loaded before loading RAPIDR. GenomicAlignments addressed the issue of the missing function *summarizeOverlaps* from GenomicRanges (bioconductor-genomicranges 1.38.0). Similar to SeqFF, RAPIDR only works with the GRCh37 human reference genome. In terms of reference creation set, the function *makeGCContentData* was used to calculate the GC content information for the GRCh37 reference. Next, the reference samples were binned using *makeBinnedCountsFile* with parameter *k* (bin size) set to *20000* (default). The samples were binned with *makeBinnedCountsFile*, and the final R object representing the reference set was made with *createReferenceSetFromCounts* with *gcCorrect* and *filterBin* set to *TRUE*. RAPIDR does output trisomy calls in addition to Z-scores, but this calling is based on the fixed Z-score cut-off threshold of three [7]. However, for uniform trisomy detection comparison over computational tools, a *Z*_*e*_ was used.

### Fetal DNA fraction calculation

For the original sequencing data and subsampled lower coverage sample sets, cell-free fetal DNA fraction (FF) was calculated using SeqFF software [21]. For SeqFF, all analysed samples were aligned against the human reference assembly GRCh37 and filtered by the alignment quality of Q30. After the filtering, the reads were sorted, and SeqFF estimates were calculated.

## Supporting information

S1 Table The number of false-negative and positive trisomy cases (with percentage) for each analysed software. (PDF)

S2 Table Summary information for each analysed sample group. The summary includes coverage group, average read count in millions of reads (M RPS), condition, coverage standard deviation, minimum, average and maximum read count and a number of samples (N).
(PDF)

S1 Fig **The percentage of false-negative (A) and false-positive (B) cases of trisomy 13 on different sequencing coverages.** (A) depicts percentages and the absolute number of false negative trisomy cases out of all known trisomy cases and (B) illustrates false-positive T13 calls out of all samples obtained with each NIPT software tool in case of various sequencing coverages. The horizontal dashed line marks the 1% cut-off, often used in case of clinical screening tests.
(TIF)

S2 Fig **The percentage of false-negative (A) and false-positive (B) cases of trisomy 18 on different emulated sequencing coverages.** (A) depicts percentages and the absolute number of false negative trisomy cases out of all known trisomy cases and (B) illustrates false-positive T18 calls out of all samples obtained with each NIPT software tool in case of various sequencing coverages. The horizontal dashed line marks the 1% cut-off, often used in case of clinical screening tests.
(TIF)

S3 Fig Effect of the natural sequencing read placement uncertainty on trisomy 21 inference. The natural sequencing read placement effect on T21 inference has a considerable effect with coverages lower than 7M RPS (0.96–24.5% of FN). With coverages 7M RPS and higher, the effect is insignificant (leads to less than 0.43% of FN T21 cases).
(TIF)

S4 Fig Effect of the natural sequencing read placement uncertainty on euploid sample group number of false-positive trisomy 21 cases. To summarise, 0.33%–0.44% of Z-scores depending on the subsample group were detected as FP T21 cases.
(TIF)

S5 Fig Chromosome 21 *Z*_*e*_ cut-off threshold variability. While for most tools the *Z*_*e*_ cut-off threshold does not change across different sequencing depths, for some tools, the empirical *Z*_*e*_ does vary considerably.
(TIF)

S6 Fig Comparison of 20M RPS fetal fraction estimates with the estimates on lower coverages. The Pearson correlation of the 20M RPS FF 5–15% estimates and the estimates on sequencing depths of 15M RPS (A), 10M RPS (B), 5M RPS (C), 2.5M RPS (D), and 1.25M RPS (E).
(TIF)

S7 Fig Fetal fraction effect on false-negative calls the of clinically validated trisomy 13 on different sequencing coverages. Computational tools were evaluated on 20 (A), 15 (B), 10 (C), 5 (D), 2.5 (E) and 1.25M RPS (F). The empirical cut-off was used for calling aneuploidy (internal classification in the case of GIPseq). Visualised samples are clinically validated T13 samples emulated to different coverages, and black triangles represent undetected aneuploidy. GIPseq was the only evaluated computational NIPT tool, which detected all 3 T13 samples on 2.5M RPS.
(TIF)

S8 Fig Fetal fraction effect on false-negative calls of the clinically validated trisomy 18 on different sequencing coverages. Computational tools were evaluated on 20 (A), 15 (B), 10 (C), 5 (D), 2.5 (E) and 1.25M RPS (F). The empirical cut-off was used for calling aneuploidy (internal classification in the case of GIPseq). Visualised samples are clinically validated T18 samples emulated to different coverages, and black triangles represent undetected trisomy.
(TIF)
