# Supplementary material for: Systematic evaluation of NIPT aneuploidy detection software tools with clinically validated NIPT samples
Source: PLoS Comput Biol. 2021 Dec 20;17(12):e1009684. doi: 10.1371/journal.pcbi.1009684 (PMC8722721; doi:10.1371/journal.pcbi.1009684)
Supplement: S1 Table — (PDF) [file pcbi.1009684.s001.pdf]

S1 Table. The number of false-negative and positive trisomy cases (with percentage) for each analysed software.

M RPS	Metric	WisecondorX	RAPIDR	NIPTmer	NIPTeR NCV	NIPTeR	GIPseq
20	T21 FN	0 (0)	3 (0.735)	0 (0)	0 (0)	0 (0)	0 (0)
20	T21 FP	1 (0.243)	16 (3.922)	1 (0.243)	2 (0.485)	1 (0.243)	1 (0.245)
15	T21 FN	0 (0)	3 (0.732)	0 (0)	0 (0)	0 (0)	0 (0)
15	T21 FP	0 (0)	9 (2.195)	0 (0)	1 (0.243)	1 (0.243)	0 (0)
10	T21 FN	0 (0)	4 (0.973)	2 (0.485)	0 (0)	0 (0)	0 (0)
10	T21 FP	1 (0.243)	10 (2.433)	1 (0.243)	1 (0.243)	2 (0.485)	0 (0)
5	T21 FN	0 (0)	4 (0.971)	2 (0.485)	0 (0)	0 (0)	0 (0)
5	T21 FP	0 (0)	8 (1.942)	0 (0)	0 (0)	0 (0)	0 (0)
2.5	T21 FN	2 (0.485)	6 (1.456)	5 (1.214)	2 (0.485)	2 (0.485)	1 (0.243)
2.5	T21 FP	0 (0)	3 (0.728)	0 (0)	0 (0)	0 (0)	0 (0)
1.25	T21 FN	5 (1.214)	16 (3.883)	9 (2.184)	4 (0.971)	4 (0.971)	3 (0.728)
1.25	T21 FP	1 (0.243)	0 (0)	1 (0.243)	0 (0)	0 (0)	1 (0.243)
20	T18 FN	0 (0)	8 (2.015)	0 (0)	0 (0)	0 (0)	0 (0)
20	T18 FP	1 (0.249)	0 (0)	0 (0)	0 (0)	NA	0 (0)
15	T18 FN	0 (0)	8 (2.005)	1 (0.249)	0 (0)	0 (0)	0 (0)
15	T18 FP	2 (0.499)	0 (0)	0 (0)	0 (0)	0 (0)	0 (0)
10	T18 FN	0 (0)	8 (2)	1 (0.249)	0 (0)	0 (0)	0 (0)
10	T18 FP	1 (0.249)	0 (0)	0 (0)	0 (0)	0 (0)	0 (0)
5	T18 FN	0 (0)	8 (1.995)	1 (0.249)	0 (0)	1 (0.249)	1 (0.249)
5	T18 FP	0 (0)	1 (0.249)	0 (0)	0 (0)	0 (0)	0 (0)
2.5	T18 FN	1 (0.249)	8 (1.995)	3 (0.748)	3 (0.748)	3 (0.748)	1 (0.249)
2.5	T18 FP	0 (0)	0 (0)	0 (0)	0 (0)	0 (0)	0 (0)
1.25	T18 FN	3 (0.748)	8 (1.995)	5 (1.247)	3 (0.748)	4 (0.998)	2 (0.499)
1.25	T18 FP	0 (0)	0 (0)	1 (0.249)	0 (0)	0 (0)	1 (0.249)
