# Supplementary material for: Systematic evaluation of NIPT aneuploidy detection software tools with clinically validated NIPT samples
Source: PLoS Comput Biol. 2021 Dec 20;17(12):e1009684. doi: 10.1371/journal.pcbi.1009684 (PMC8722721; doi:10.1371/journal.pcbi.1009684)
Supplement: S2 Table — The summary includes coverage group, average read count in millions of reads (M RPS), condition, coverage standard deviation, minimum, average and maximum read count and a number of samples (N). (PDF) [file pcbi.1009684.s002.pdf]

S2 Table. Summary information for each analysed sample group. The summary includes coverage group, average read count in millions of reads (M RPS), condition, coverage standard deviation, minimum, average and maximum read count and a number of samples (N).

M RPS	Sample Group	SD	Min	Average	Max	N
20	T21 (clinically validated)	4,277,096	14,347,516	24,163,449	32,626,785	19
15	T21 (clinically validated)	3,148,835	10,937,064	18,083,151	24,334,564	19
10	T21 (clinically validated)	2,081,671	7,209,906	11,958,958	16,127,678	19
5	T21 (clinically validated)	1,048,024	3,670,427	6,023,952	8,173,000	19
2.5	T21 (clinically validated)	523,966	1,833,823	3,011,415	4,086,277	19
1.25	T21 (clinically validated)	262,260	915,450	1,505,824	2,042,374	19
20	T18 (clinically validated)	2,791,633	18,450,798	23,124,624	27,336,184	8
15	T18 (clinically validated)	2,071,817	14,024,066	17,350,621	20,485,890	8
10	T18 (clinically validated)	1,395,351	9,235,616	11,476,668	13,625,081	8
5	T18 (clinically validated)	697,871	4,685,815	5,794,650	6,891,991	8
2.5	T18 (clinically validated)	348,455	2,344,985	2,898,169	3,446,589	8
1.25	T18 (clinically validated)	174,390	1,172,245	1,449,377	1,722,745	8
20	T13 (clinically validated)	2,980,546	19,042,280	22,183,555	24,971,957	3
15	T13 (clinically validated)	2,257,362	14,500,538	16,725,422	19,013,917	3
10	T13 (clinically validated)	1,469,534	9,567,867	11,039,287	12,506,927	3
5	T13 (clinically validated)	742,824	4,870,521	5,591,835	6,354,446	3
2.5	T13 (clinically validated)	370,783	2,435,984	2,796,437	3,176,752	3
1.25	T13 (clinically validated)	184,533	1,219,133	1,398,883	1,587,854	3
20	Validation Low Risk	3,594,972	13,643,477	23,747,773	32,396,033	393
15	Validation Low Risk	2,688,192	10,345,586	17,800,264	24,374,332	393
10	Validation Low Risk	1,791,357	6,839,734	11,799,169	16,181,461	393
5	Validation Low Risk	896,917	3,453,849	5,944,443	8,061,718	393
2.5	Validation Low Risk	448,456	1,726,568	2,972,178	4,028,665	393
1.25	Validation Low Risk	224,239	863,244	1,486,192	2,014,776	393
20	Reference	6,032,848	11,534,450	23,506,647	55,026,706	669
15	Reference	4,531,992	8,656,925	17,654,442	41,383,148	669
10	Reference	3,013,960	5,736,648	11,695,518	27,432,704	669
5	Reference	1,516,731	2,919,971	5,942,981	13,920,933	669
2.5	Reference	758,325	1,458,860	2,971,523	6,957,681	669
1.25	Reference	379,135	729,274	1,485,737	3,476,940	669
