# Supplementary figures and images for: Systematic evaluation of NIPT aneuploidy detection software tools with clinically validated NIPT samples
Source: PLoS Comput Biol. 2021 Dec 20;17(12):e1009684. doi: 10.1371/journal.pcbi.1009684 (PMC8722721; doi:10.1371/journal.pcbi.1009684)

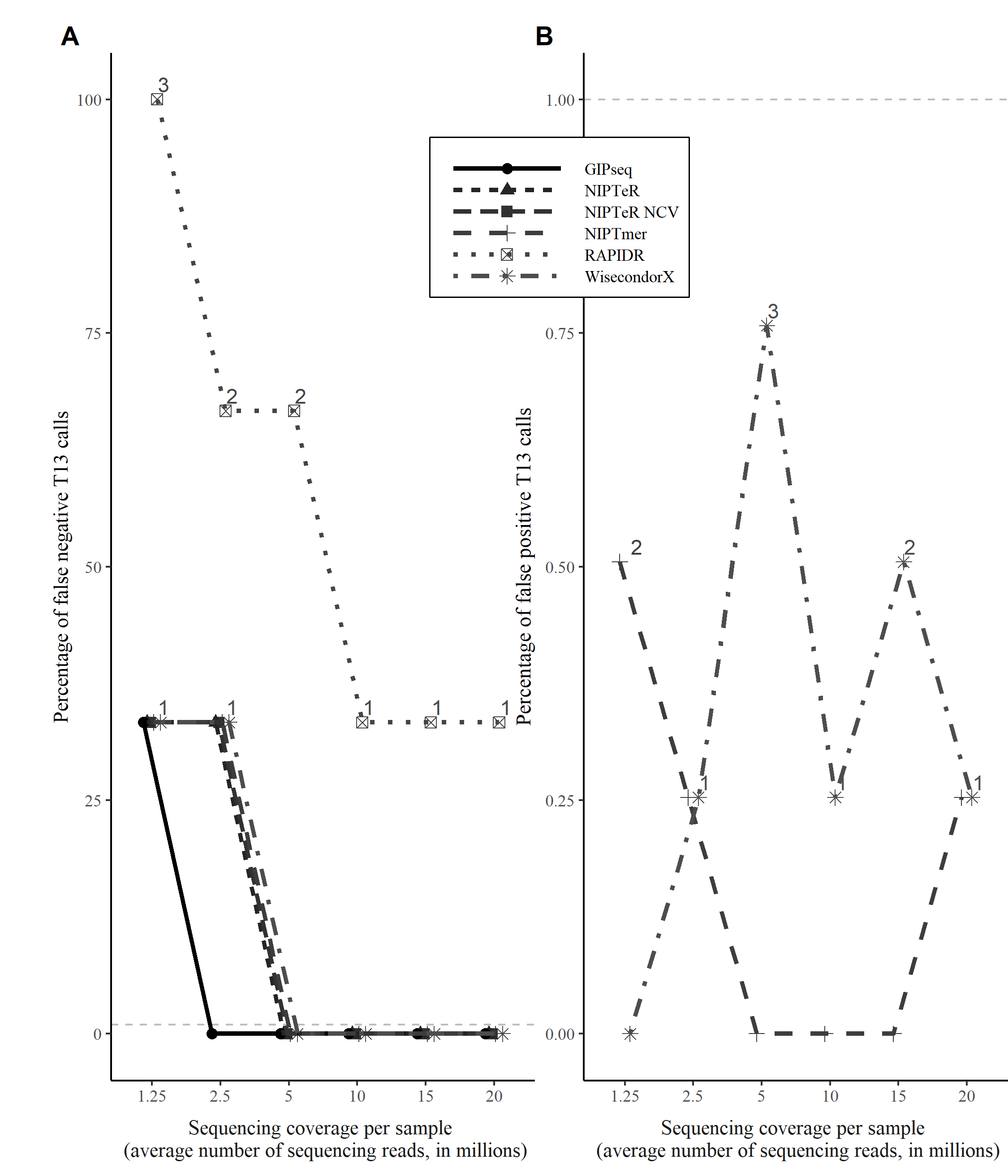

Supplement: S1 Fig — The percentage of false-negative (A) and false-positive (B) cases of trisomy 13 on different sequencing coverages. (A) depicts percentages and the absolute number of false negative trisomy cases out of all known trisomy cases and (B) illustrates false-positive T13 calls out of all samples obtained with each NIPT software tool in case of various sequencing coverages. The horizontal dashed line marks the 1% cut-off, often used in case of clinical screening tests. (TIF) [file pcbi.1009684.s003.tif]

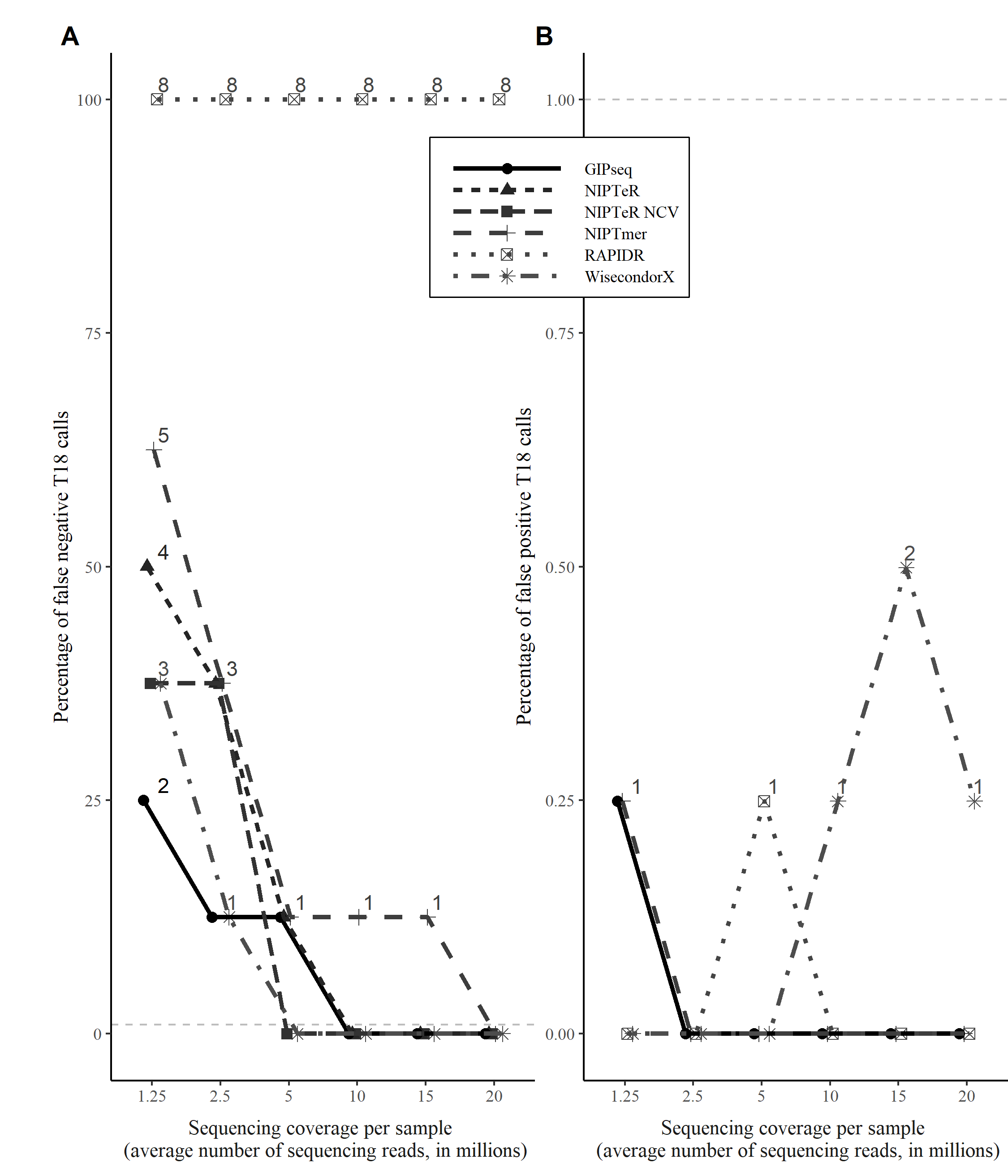

Supplement: S2 Fig — The percentage of false-negative (A) and false-positive (B) cases of trisomy 18 on different emulated sequencing coverages. (A) depicts percentages and the absolute number of false negative trisomy cases out of all known trisomy cases and (B) illustrates false-positive T18 calls out of all samples obtained with each NIPT software tool in case of various sequencing coverages. The horizontal dashed line marks the 1% cut-off, often used in case of clinical screening tests. (TIF) [file pcbi.1009684.s004.tif]

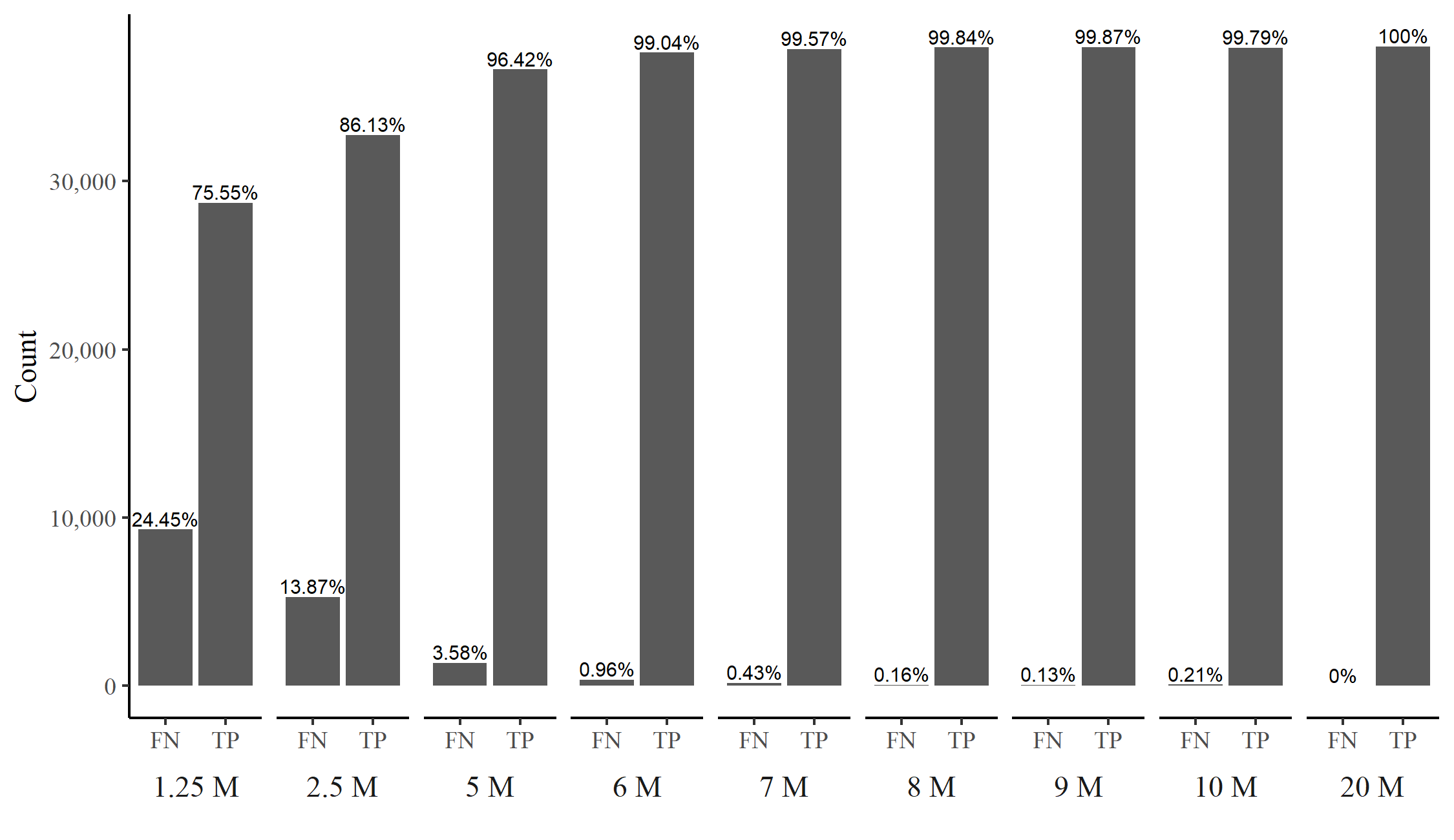

Supplement: S3 Fig — The natural sequencing read placement effect on T21 inference has a considerable effect with coverages lower than 7M RPS (0.96–24.5% of FN). With coverages 7M RPS and higher, the effect is insignificant (leads to less than 0.43% of FN T21 cases). (TIF) [file pcbi.1009684.s005.tif]

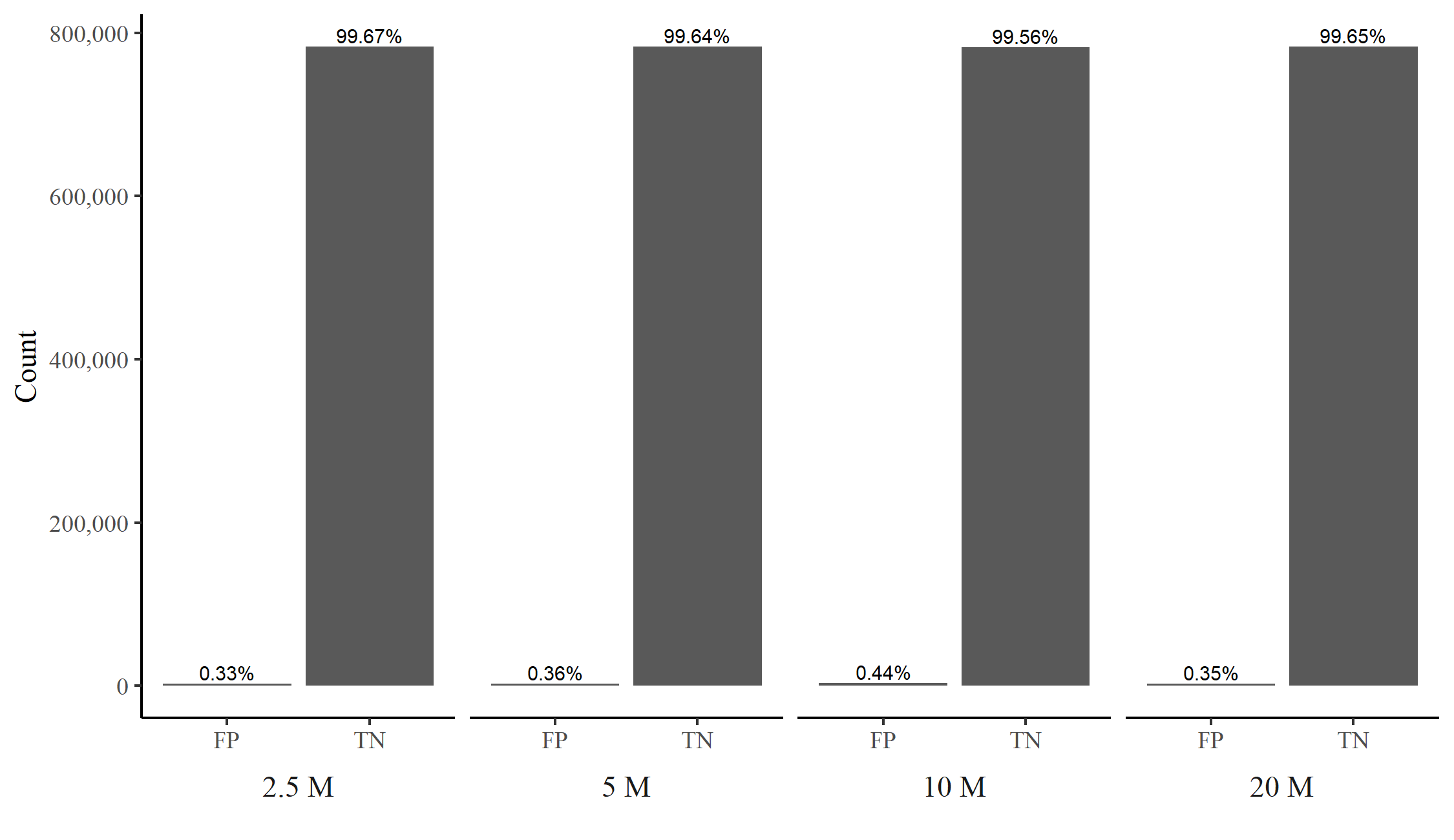

Supplement: S4 Fig — To summarise, 0.33%–0.44% of Z-scores depending on the subsample group were detected as FP T21 cases. (TIF) [file pcbi.1009684.s006.tif]

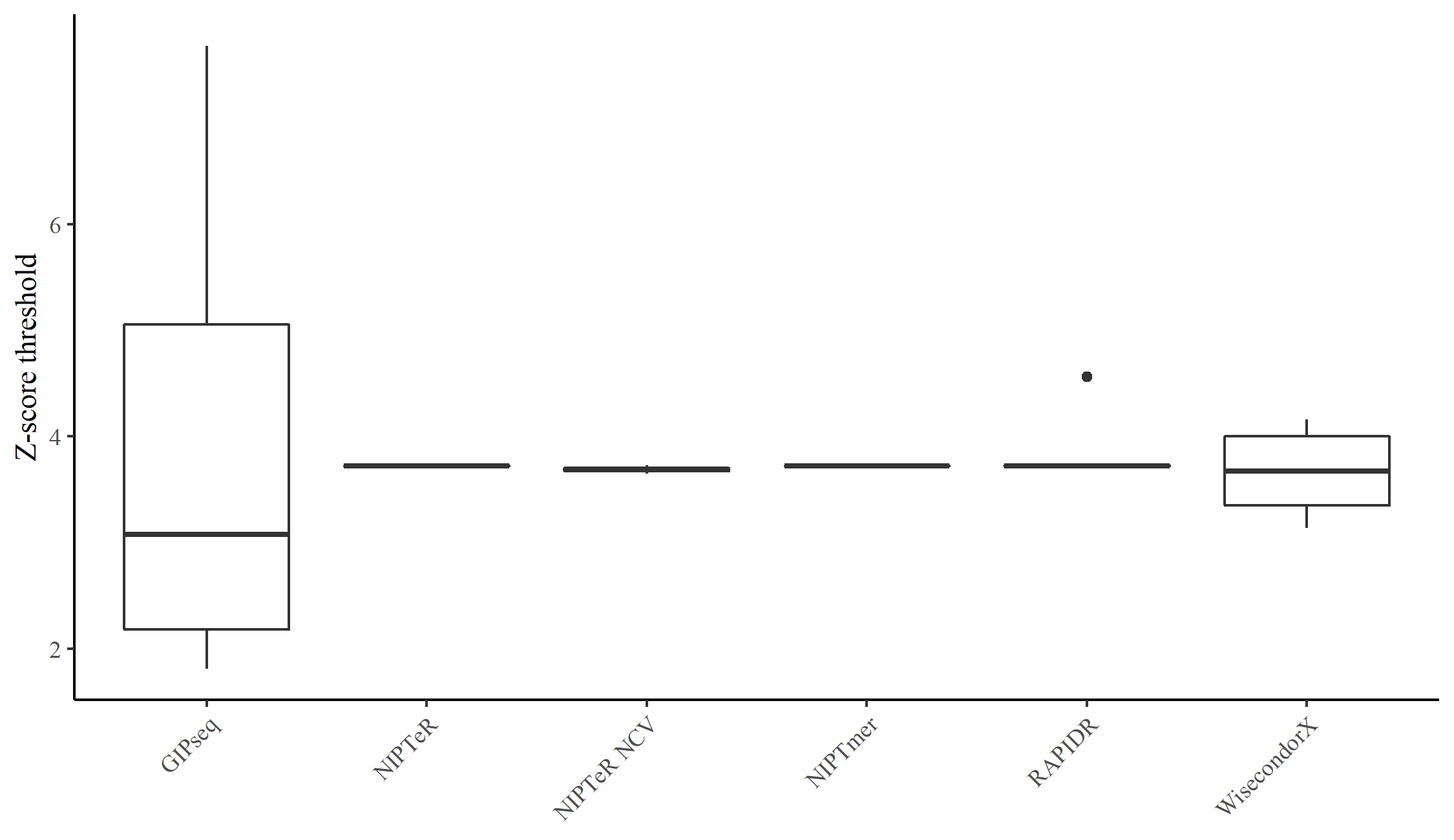

Supplement: S5 Fig — While for most tools the Ze cut-off threshold does not change across different sequencing depths, for some tools, the empirical Ze does vary considerably. (TIF) [file pcbi.1009684.s007.tif]

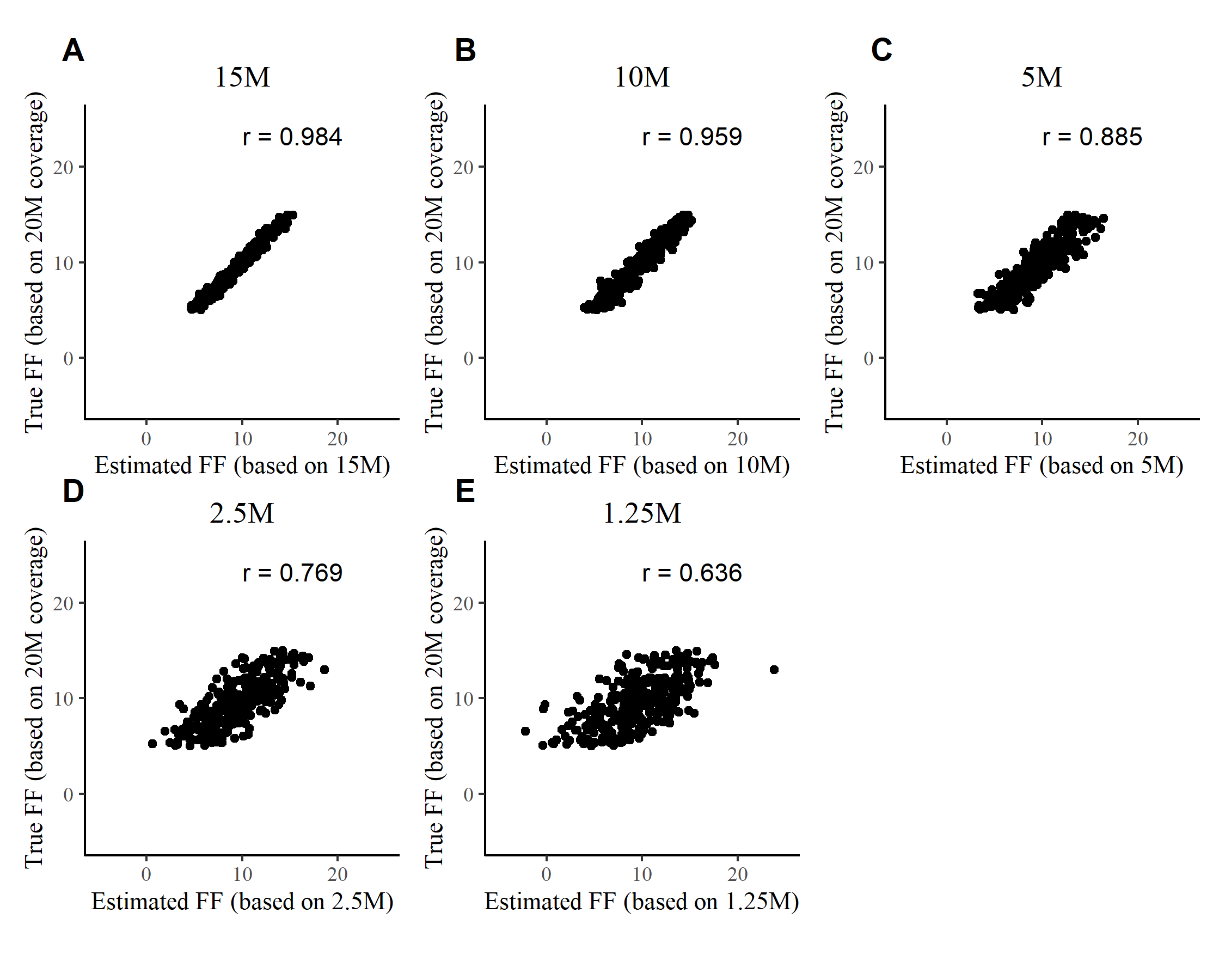

Supplement: S6 Fig — The Pearson correlation of the 20M RPS FF 5–15% estimates and the estimates on sequencing depths of 15M RPS (A), 10M RPS (B), 5M RPS (C), 2.5M RPS (D), and 1.25M RPS (E). (TIF) [file pcbi.1009684.s008.tif]

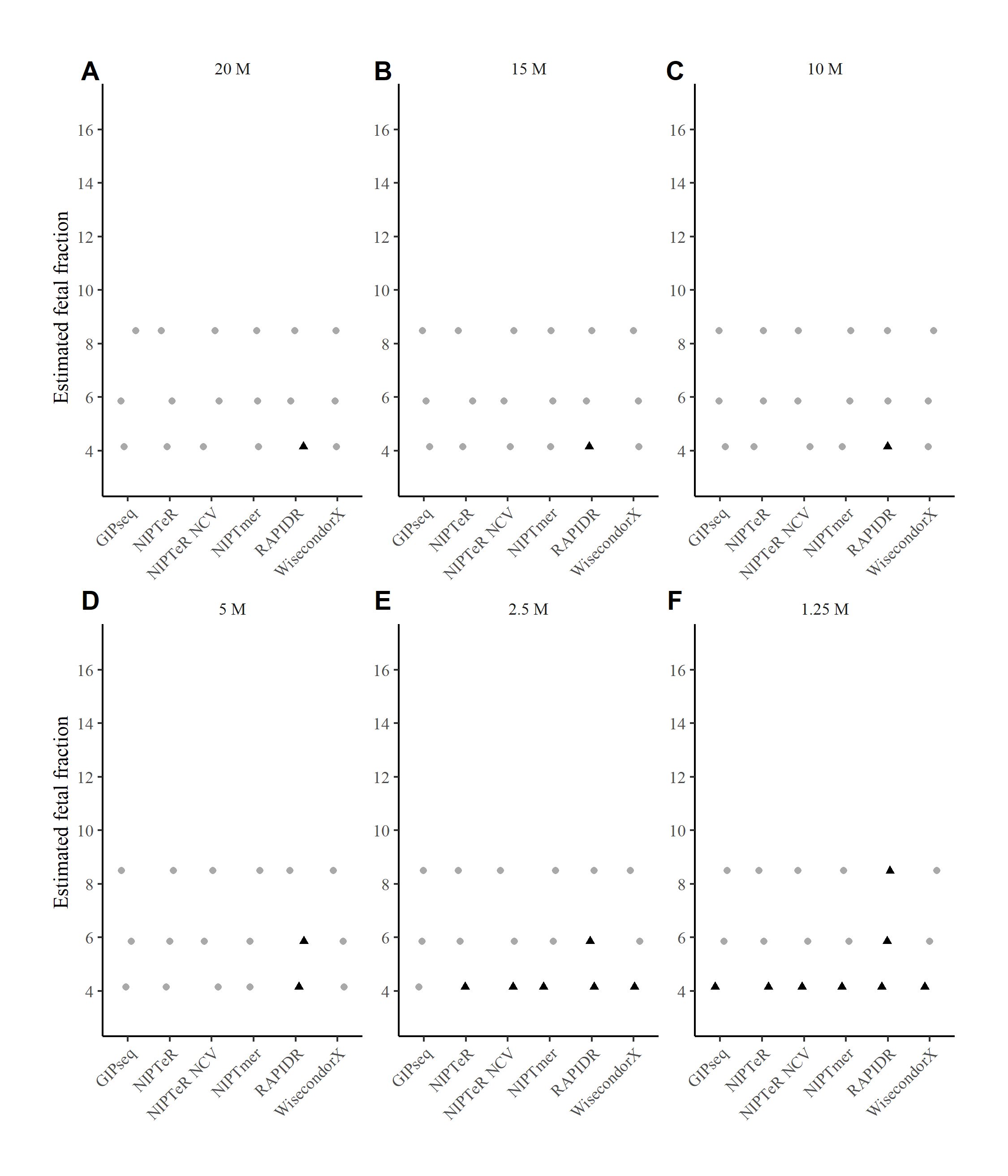

Supplement: S7 Fig — Computational tools were evaluated on 20 (A), 15 (B), 10 (C), 5 (D), 2.5 (E) and 1.25M RPS (F). The empirical cut-off was used for calling aneuploidy (internal classification in the case of GIPseq). Visualised samples are clinically validated T13 samples emulated to different coverages, and black triangles represent undetected aneuploidy. GIPseq was the only evaluated computational NIPT tool, which detected all 3 T13 samples on 2.5M RPS. (TIF) [file pcbi.1009684.s009.tif]

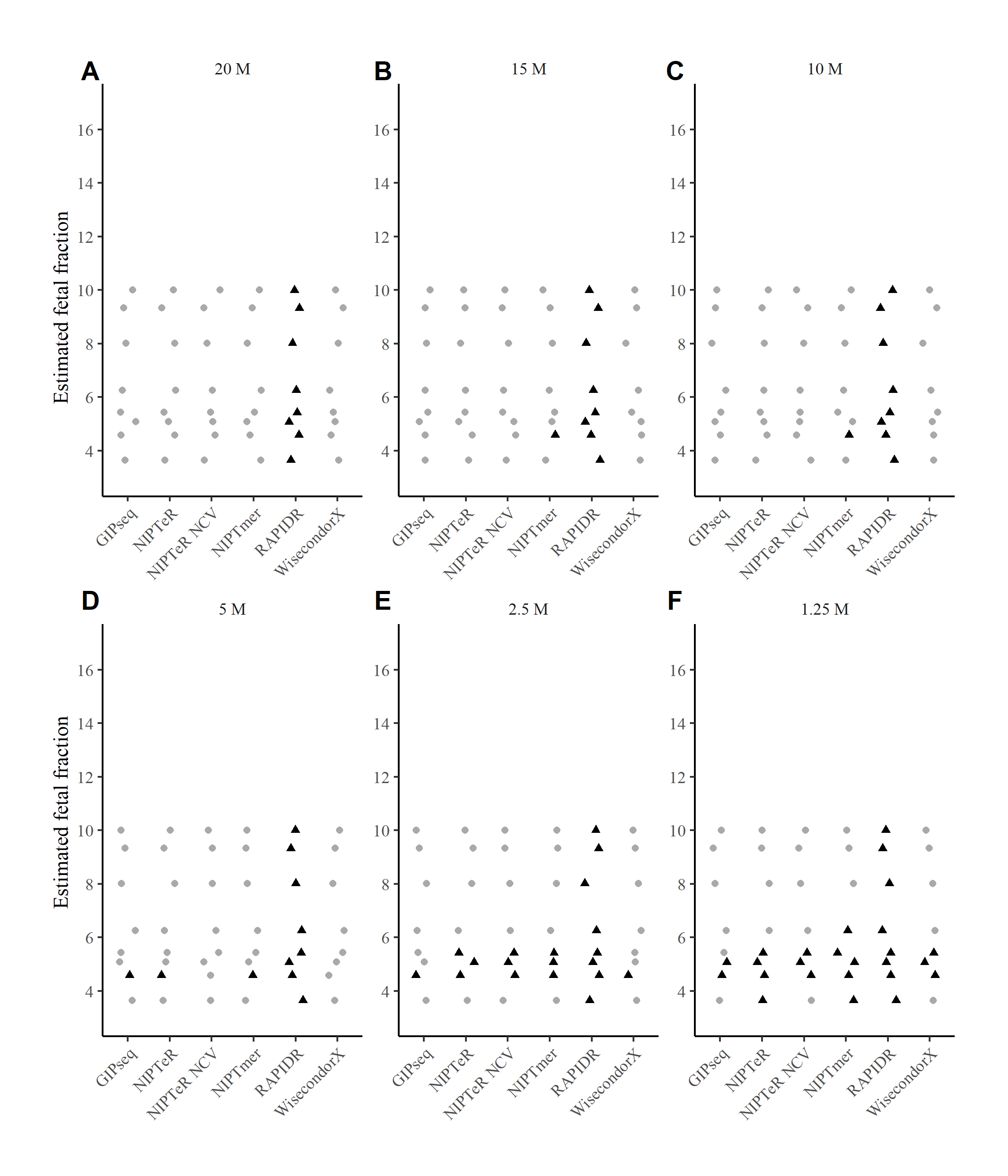

Supplement: S8 Fig — Computational tools were evaluated on 20 (A), 15 (B), 10 (C), 5 (D), 2.5 (E) and 1.25M RPS (F). The empirical cut-off was used for calling aneuploidy (internal classification in the case of GIPseq). Visualised samples are clinically validated T18 samples emulated to different coverages, and black triangles represent undetected trisomy. (TIF) [file pcbi.1009684.s010.tif]
